# Exploring the inherent mechanism of residents’ participation behavior in neighborhood regeneration projects: an empirical study using an extended IMB model in China

**DOI:** 10.3389/fpsyg.2023.1257970

**Published:** 2023-11-09

**Authors:** Xinyue Fu, Taozhi Zhuang, Ruopeng Huang, Yaxian Dong

**Affiliations:** ^1^School of Management Science and Real Estate, Chongqing University, Chongqing, China; ^2^Management in the Built Environment, Faculty of Architecture and the Built Environment, Delft University of Technology, Delft, Netherlands; ^3^Engineering Unit A, Department of Architectural Engineering, The Pennsylvania State University, University Park, PA, United States

**Keywords:** residents’ participation behavior, inherent mechanism, neighborhood regeneration, information-motivation-behavioral model, structural equation modeling

## Abstract

**Introduction:**

Resident participation has gained increasing prominence and significance in the pursuit of sustainable neighborhoods regeneration. However, the current state of resident participation practices remains beset by several challenges, which present formidable impediments to the initiation and execution of neighborhood regeneration initiatives. This study aims to investigate the underlying mechanisms of residents’ participation behavior to enhance resident participation in neighborhood regeneration projects.

**Methods:**

The present study employs the extended Information-Motivation-Behavioral (IMB) model to examine the determinants and mechanisms influencing residents’ willingness and participation in neighborhood regeneration projects, with a specific focus on the Chinese context. Drawing upon data from 477 meticulously validated questionnaires administered to residents, the study applies structural equation modeling (SEM) to unravel the intrinsic dynamics of residents’ participation behavior.

**Results:**

The empirical findings of this research reveal that information, motivation, and the perceived local government support all exert a significant impact on residents’ participation willingness. Notably, motivation emerges as the most influential factor.

**Discussion:**

This study uncovers a direct influence of local government organizations on both residents’ willingness and their actual participation, suggesting that government organizations can spearhead innovative strategies to bolster residents’ willingness and furnish avenues for translating willingness into tangible participation. The outcomes of this study furnish an indispensable theoretical framework and offer policy recommendations that hold paramount importance for the deployment of novel interventions geared toward stimulating active involvement of residents in neighborhood regeneration.

## 1. Introduction

There is growing global interest in neighborhood regeneration as it is seen as a way to improve the urban environment and enhance the welfare of citizens (Huang et al., 2023a,b,c). Neighborhood regeneration, a vital component of urban development, is closely linked to the achievement of the New Urban Agenda and the Sustainable Development Goals proposed by the United Nations which are geared toward achieving a better and more sustainable future for all [United Nations (UN), 2015]. Many papers highlight global trends in improving neighborhoods, showcasing projects happening worldwide. For instance, the Dutch government launched the so-called Big City Policy, which included measures presented in the Urban Regeneration Memorandum aimed at preventing gentrification and physical decline in neighborhoods (Teernstra and Pinkster, 2016). In the UK, the “Neighborhood Renewal-People and Place” campaign was launched to bridge the gap between the quality of life in the poorest neighborhoods and the rest of society [Neighborhood renewal (NR), 2021]. Similarly, in China, around 170,000 dilapidated neighborhoods, covering a floor area of 800 million m^2^ and affecting over 42 million households, are in need of regeneration (Liu B. et al., 2022). As such, the implementation of neighborhood regeneration is a crucial global issue (Huang et al., 2023a,b,c).

As with all multidimensional and complex public issues, neighborhood regeneration projects are closely linked to residents’ life. Variously, the unique nature of the neighborhood regeneration projects has led to a greater need for extensive public participation, particularly from local residents, than other public issues (Norris and Hearne, 2016; Fu et al., 2023). In some general public affairs, decisions are usually made by the government or some elite coalition, and residents are usually left out of the decision-making process. However, the situation in the neighborhood regeneration projects is quite different, where residents play an important and irreplaceable role. As the residents involved in neighborhood regeneration projects were already living here before the project started, they will be the decision-makers and witness the whole process of regeneration. Meanwhile, they are also the end-users of this neighborhood regeneration project since they will still be living here in the future. As a result, residents’ perceptions of neighborhood regeneration projects may more sensitive than those of other public issues (such as urban traffic, park greening, etc.), as both the process and the outcome of the project are closely linked to their daily lives (Masoumi, 2019).

Encouraging resident participation in neighborhood regeneration projects is particularly significant in the Chinese context. First, in terms of geographical condition, unlike most open-plan neighborhoods in the West, Chinese neighborhoods are relatively closed and independent, which means that the regeneration affairs of this neighborhood are closely linked to the residents who live there. In other words, promoting the participation of this particular group of residents has a crucial impact on the success of neighborhood regeneration projects. Second, the promotion of resident participation is increasingly influenced by the ideological ideas of “good governance” and democratic policy-making (Lawson and Kearns, 2010). However, unlike the long-established and mature public participation mechanisms in the West, the active participation of Chinese residents can sometimes lead to social conflict (Li J. et al., 2020; Huang et al., 2023a,b,c). Historically, urban development decisions in China have been made by the government, excluding residents from the decision-making process. As a result, residents either passively accept the outcome of decisions or engage in group conflict to pursue their interests (Liu G. et al., 2022). Therefore, it is important to explore ways to guide residents to participate actively and orderly throughout the entire neighborhood regeneration process.

In addition, from the perspective of the Chinese government, promoting resident participation is also essential. Many cities in China have established minimum resident requirements for starting neighborhood regeneration projects [The People’s Government of Shantou Municipality (PGSM), 2014; The People's Government of Guangzhou Municipality (PGGM), 2020]. On the one hand, the government hopes that residents will contribute to alleviating the financial pressure on the long-term operation of the project, while on the other hand, the call for residents’ participation is fundamentally an echo of the administrative decentralization proposed by the government in recent years. The government wants to change from being the manager in charge of everything to being the coordinator who only provides a platform for the participation of the residents. The successful implementation of this change in role is predicated on extensive resident participation. Therefore, the issue of resident participation in the neighborhood regeneration projects in China has its own unique research value, both from the perspective of the residents (geo-environmental and participatory awareness) and from the perspective of the government (political requirements and governance needs).

Exploring the mechanisms of resident participation and its impact on neighborhood regeneration projects is quite crucial to increasing public participation, which has attracted the attention of many scholars (Wu et al., 2020; Chamusca, 2023). However, it has proved very complicated to open the “black box” (Conrad et al., 2011). Residents’ participation behaviors are somehow stochastic and uncertain, influenced by both individual factors, social factors, and external factors. Although some studies have tried to examine the factors influencing residents’ participation behavior from the individual (i.e., information received by residents, participation willingness or behavior skills) or social (i.e., interaction between residents) perspective (Hilvert-Bruce et al., 2018; Dessart et al., 2019; Ye et al., 2022), there is still a lack of a unified framework for considering all these factors in a holistic manner. The information-motivation-behavioral (IMB) model, a well-established model in the behavioral sciences that explains the influence of internal personality and interpersonal relationships on human behavioral decisions, provides a viable research framework for addressing this issue. In addition, the characteristics of Chinese neighborhood regeneration projects (especially the interaction between government and residents) are used as a supplement to make this model more applicable to the research scenario of this paper. Therefore, this study applied the extend IMB model to analyze the resident participation behaviors.

The research in this paper aims to explore the mechanism of resident participation in neighborhood regeneration projects in China. It answers: What are the influencing factors? How do these factors shape the residents’ participation behavior? What are effective ways to promote resident participation in neighborhood regeneration projects? As a representative city in Southwest China, Chongqing has a great deal of experience in regeneration and was therefore chosen as the case city for this study. The rest of the paper is organized as follows. The remainder of the paper is structured as follows. Section 2 encompasses a review of existing literature and the formulation of the research hypothesis. Section 3 provides detailed information on the data and methodology. Section 4 shows the results, and Section 5 offers a discussion. Finally, Section 6 summarizes the entire study.

## 2. Literature review and hypotheses development

### 2.1. Inherent mechanism of participation behavior

In order to study the mechanism of residents’ behavior, two aspects should be clarified. One is to explore the influencing factors and the other is to clarify the influencing path (Liu et al., 2021). Some scholars have proposed that having the participation willingness is an important prerequisite for residents to implement the participation behavior (Zheng et al., 2023). Further, some studies focus on which factors affect willingness, so as to analyze the complete influencing path from influencing factors to participation behavior (through the transmission of participation willingness; Wang S. et al., 2017).

There exist many studies probing the influencing factors of residents’ behavioral willingness from diverse perspectives. Many scholars have studied the influencing factors from individual perspective (Dessart et al., 2019). Individual factors relate to an individual’s general propensity to behave in a certain way (Malle, 2011). The information available to decision-makers may have an impact on their behavioral decisions, and different information may introduce behavioral biases (Hsieh and Lo, 2021). Besides, individual factors are internal variables related to a given individual, such as behavioral willingness, general preferences, behavioral skills, motivations, values, and expectations (Palmer et al., 2020; Yang et al., 2020).

In addition to individual factors, the influence of social factors on residents’ willingness has also been widely discussed. Social factors are related to residents’ interactions with others, of which social motivation are the most frequently discussed (Li et al., 2022; Ye et al., 2022). Many researchers have conceptualized how social motivations affect people’s willingness and behaviors in different ways. Some scholars argue that motivation is the basic driver of people’s social behavior (Hilvert-Bruce et al., 2018), while others point out that social motivation is an intrinsic factor that facilitates individuals’ activities to achieve certain goals (Hernandez et al., 2011). Residents’ motivation may be driven by perceived social pressures from the group, which may come from family, peers or neighbors (Zhang et al., 2015). They may comply with others’ opinions or actions, or not, but there is no doubt that it is difficult for them to make decisions outside of their own social environment (Wang C. et al., 2018).

The factors influencing participation willingness and behavior, whether individual or social factors, are explored from the inside of resident group. In contrast, some studies believe that the external factors outside the resident group (e.g., the macro environment of policies, the role of government, the influence of other stakeholders, etc.) also has an important impact on residents’ willingness and behaviors. Quantitative research on the impact of government roles on specific residents’ behaviors has been conducted in many research fields such as waste management (Benito et al., 2021), e-participation (Coelho et al., 2022), public services (Buntaine et al., 2021), etc. However, there is still a lack of relevant research in the urban field. Although it has been noted that the behavioral decisions of government organizations and residents are mutually influential in the regeneration process (Liu et al., 2020; Cao, 2022), fewer studies have expressed the influence in a quantitative manner. Therefore, there is an urgent need to construct a theoretical model that integrates internal and external factors to predict residents’ behavior and to quantify its underlying mechanisms.

### 2.2. Equations the original IMB model

**Figure 1:**
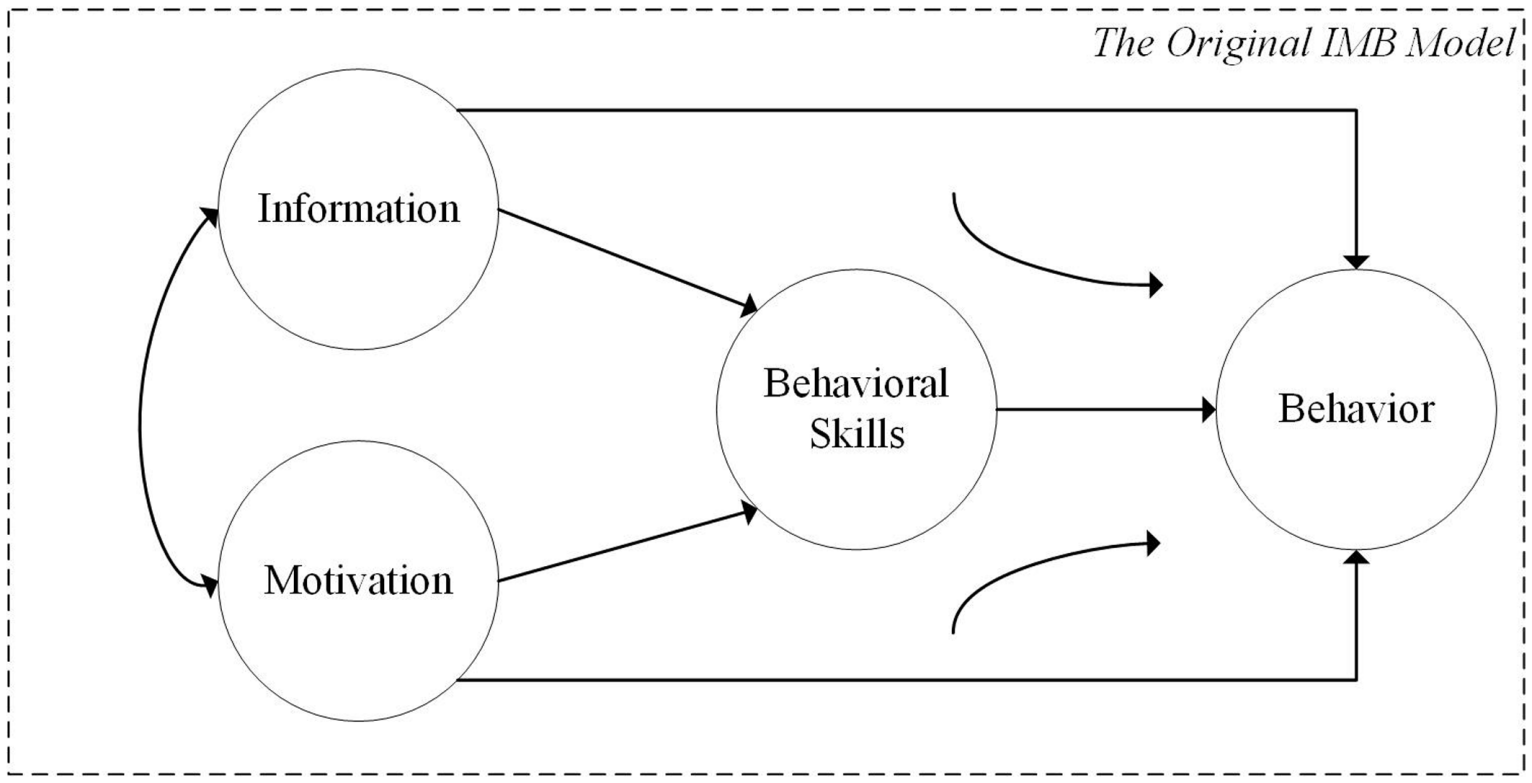
The original IMB model.

Research that combines the urban fields and behavioral science has become increasingly popular. A number of rational-choice models are being used to explain the decision-making processes of residents in relation to specific behaviors, and the IMB model is one of these. This model, first proposed by Fisher and Fisher (1992), is a comprehensive model to predict behavior change, which is used to explain the influence of external environment and interpersonal relationships on human behavior decisions. The original IMB model is shown in Figure 1, and it distinguishes three core components that influence participation behavior: information, personal and social motivation, and behavioral skills (Fisher et al., 2003).

*Information* is accurate, behavior-specific knowledge (Tsamlag et al., 2020). In other words, information refers to whether an individual has accurate information that can be translated into participating behaviors (Wang X. et al., 2018). Therefore, in this paper, the information component specifically refers to residents’ cognition of neighborhood regeneration and an accurate understanding of its consequences.

*Motivation* is defined as the internal driving force or goal that guides and motivates individuals to engage in a specific behavior (Michie et al., 2008). Motivation-dependent interventions are often based on social norms, group identity, or other tools appropriate to the context of the intervention (e.g., commitment to action, intention to implement, effect of resident leaders; Park and Kwon, 2017; Hodges et al., 2020). These interventions can influence attention and the ease of thinking about related concepts, even if they don’t alter a person’s fundamental motivation. For example, a message that a resident’s neighbors all are actively involved in neighborhood regeneration may make one’s pro-social motivations more accessible, encouraging conformance with the social norms of participating in the day-to-day affairs provided in the message (Huang et al., 2023a,b,c).

*Behavioral skills* include individual self-efficacy and abilities to perform certain behavior patterns (Chen et al., 2017). It reflects the individual’s goal and the perceived ability to participate in the target behavior (Hodges et al., 2020). In this paper, behavioral skills mainly refer to residents’ abilities to solve the problems encountered in neighborhood regeneration projects, such as the ability to transform information into actual participation behavior, or interpersonal skills, that is, using existing interpersonal relationships to help solve new problems encountered during the regeneration process.

According to the IMB model, the information residents have about their participation, the motivation to perform based on that information, and the behavioral skills required to conduct that behavior will determine their eventual participation behaviors (Shrestha et al., 2016). When the skills required to implement the behaviors are complex (e.g., to develop a cooperative relationship with others), the information and motivation affect the behavior mainly through the mediating role of behavioral skills. However, information and motivation may directly influence behavior when only basic skills are required to perform the participation behavior (e.g., passive acceptance of neighborhood regeneration progress; Fleary et al., 2020).

### 2.3. The extended IMB model for this research

Although the original IMB model has been widely used in behavioral prediction research, especially in the field of health and hygiene (Fleary et al., 2020; Pollard et al., 2021; Knox et al., 2022), it is still necessary to modify and develop the model to improve its explanatory ability (Tsamlag et al., 2020). Some scholars have adjusted the constructs of the IMB model by integrating, adding, or replacing them, taking into account the unique attributes of their research field. For example, Samadaee Gelehkolaee et al. (2021) proposed that “behavioral skills” can be divided into two themes: “the need for adolescent sexuality socialization management” and “the need for enhancing the teachers’ professional competence.” Tsamlag et al. (2020) redefined “motivation,” an original construct of the IMB model, as “health belief” and added a new construct, namely “medical system support” at the same time. Comparing the two aforementioned studies, it becomes apparent that their commonality resides in the alterations applied to the original constructs of the IMB model. Samadaee Gelehkolaee et al. (2021) employed a bifurcation approach, while Tsamlag et al. (2020) chose to revise an existing structure and introduce a new one. Regardless of the form of these changes, these modifications significantly enhance the applicability and explanatory power of the IMB model in various domains. These adaptable methods, which serve as valuable references, also lay the foundation for this study’s adjustments to the original IMB model, making it more suitable for research scenarios related to neighborhood regeneration.

When discussing neighborhood regeneration, we believe that if “behavioral skills” are modified as “participation willingness,” it may further promote the explanatory ability of the IMB model. This is mainly because neighborhood regeneration and urban construction in China have long been government-led, which means that behavioral skills needed by residents (e.g., collecting related information, determining the regeneration content, reaching consensus) have been taken over by the government for a long time (Guo, 2020). Thus, behavioral skills maybe not be the most important factor since even when informed and motivated, residents may still not know how to participate effectively in the neighborhood regeneration projects (Hodges et al., 2020). In the current Chinese context, the key to promoting residents’ participation behavior is whether residents are “willing” rather than “able” to do so (Tan et al., 2019). Participation willingness, vice versa, emphasizing residents’ psychological evaluation of participating behaviors, has been proved by many studies to have a direct impact on decision-makers’ behaviors.

In addition, the original IMB model emphasizes the influence of internal and interpersonal relationships on residents’ behavioral decisions, so information and motivation are considered the main influencing factors (Wang C. et al., 2018; Hodges et al., 2020). However, those two factors were selected from within the resident group, without taking the macro-environment outside the resident group into account. More and more studies show that external macro-environmental factors (e.g., policy factors, the role of local government organizations, etc.) can also guide residents’ behaviors (Cheng et al., 2020; Samadaee Gelehkolaee et al., 2021). In the implementation of neighborhood regeneration in China, local government organizations have consistently played a dual role. They act as the ultimate government organization, and on the other hand, they undertake the task of contacting with the residents face-to-face. In other words, the local government organizations are the link and bridge between the higher levels of government and the residents (Tang, 2020). They provide residents with the most direct window to contact the macro policy environment, so it is necessary to explore their role and effectiveness in promoting residents’ participation behaviors.

**Figure 2:**
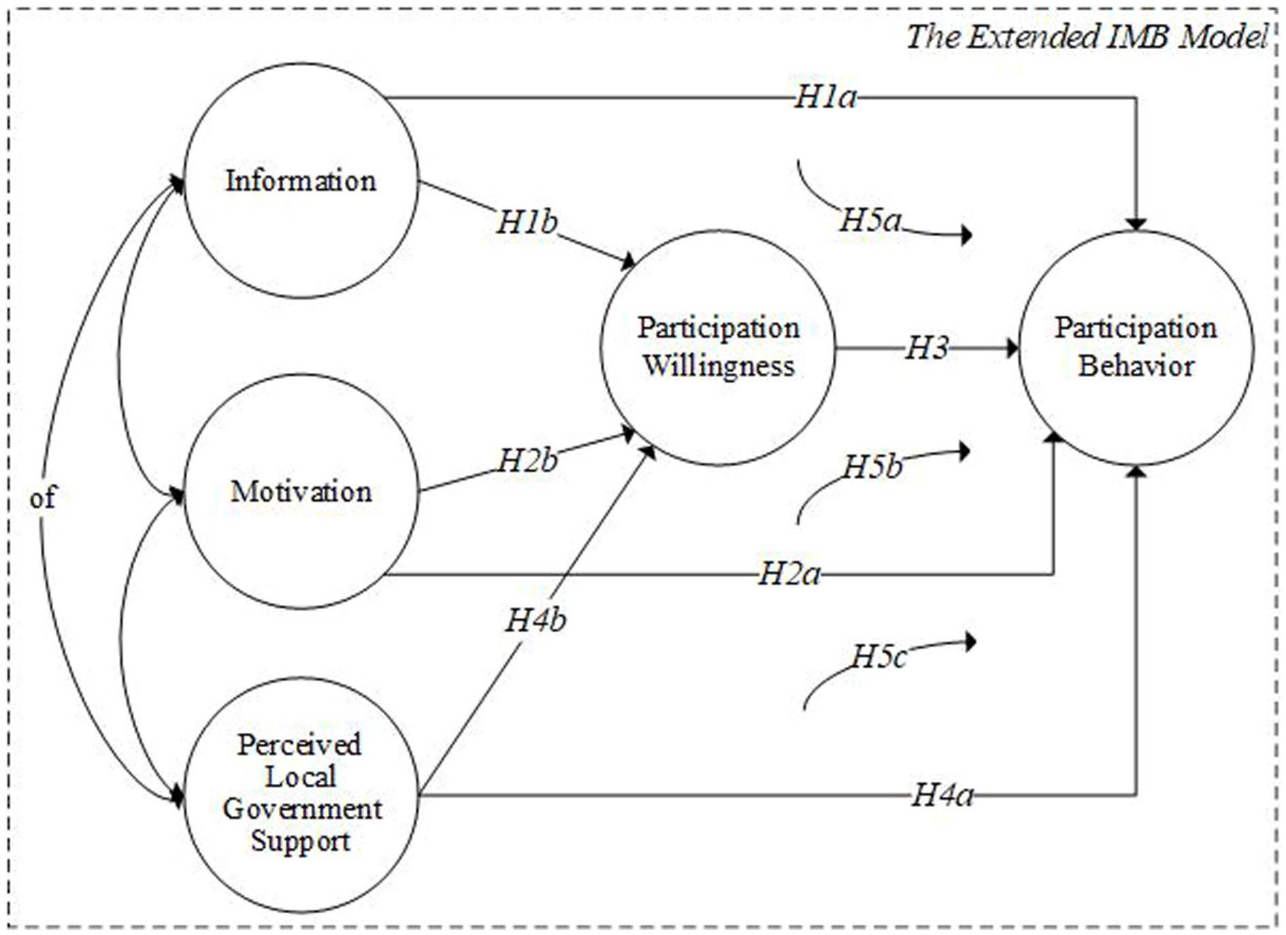
The extended IMB model for this research.

Based on the above analysis, we extended the IMB model to make it more in line with the reality of neighborhood regeneration in China. The expanded model is shown in Figure 2, and corresponding hypotheses can be put forward as follows:

H1a. Residents who possess a greater amount of information related to regenerating the physical environment are more inclined to engage in neighborhood regeneration projects.

H1b. The greater the amount of information a resident possesses regarding the regeneration of the physical environment, the more willing they are to participate in neighborhood regeneration projects.

H2a. The more motivation an in-situ resident receives, the more likely s/he participates in the neighborhood regeneration projects.

H2b. The more motivation an in-situ resident receives, the higher her/his participation willingness in the neighborhood regeneration projects.

H3. The more participation willingness a resident possesses, the more likely s/he participates in the neighborhood regeneration projects.

H4a. The greater the role played by the local government organization, the more likely residents to participate in the neighborhood regeneration projects.

H4b. The greater the role played by the local government organization, the higher the willingness of residents to participate in the neighborhood regeneration projects.

H5a. Information related to regenerating the physical environment will affect residents’ participation behavior indirectly through participation willingness.

H5b. Motivation will affect residents’ participation behavior indirectly through participation willingness.

H5c. The role of the local government organization will affect residents’ participation behavior indirectly through participation willingness.

## 3. Research methods and data

### 3.1. Study setting

Chongqing’s neighborhood regeneration and urban development model are representative of China (Huang et al., 2020). This research, focusing on Chongqing as the study area, involved a questionnaire survey carried out in 68 old neighborhoods situated within 7 districts of Chongqing. Over the past few decades, Chongqing has experienced rapid urbanization. Based on data from 2023, the total area in Chongqing had expanded to around 1,200 square kilometers, with an estimated permanent population of nearly 7.2 million people. More than 50 million m^2^ of houses have been used for more than 20 years (Liu et al., 2021). The outdated urban function of the old neighborhoods is increasingly unable to meet the demand of its residents for a better living environment. In order to cure the “urban disease” and improve the life quality, Chongqing Municipal People’s Government has proposed to take the lead in promoting neighborhood regeneration. Since 2021, Chongqing has been implementing a neighborhood regeneration program, covering 831 dilapidated neighborhoods and involving the construction of 26.62 million square meters of urban space. By the end of 2022, Chongqing had initiated the regeneration of 3,993 old neighborhoods, which covered 92.27 million square meters and benefited 990,000 households (Chongqing Housing and Urban-Rural Development Commission, 2022). The extensive history of regeneration practices in Chongqing has furnished our study with vital and diverse research materials.

### 3.2. Questionnaire design and measurement instruments

The questionnaire was created using the research framework and hypotheses we mentioned earlier. It comprises two parts: the first part includes general demographic questions, while the second part comprises constructs and items relevant to this study. All constructs were measured using multi-item scales, which were based on general recommendations from Francis et al. (2004) and previous research on neighborhood regeneration and residents’ participation behaviors (refer to Table 1). Seven-point Likert scale was utilized to measure item scores, where 1 indicates “strongly disagree” and 7 indicates “strongly agree.”

**Table 1:** Measures of constructs.

Construct	Item	Question	References
Information	INF1	I know the latest news or subsidy regulations for the neighborhood regeneration project.	Tan et al. (2020), Tsamlag et al. (2020), and Wang et al. (2021)
	INF2	I learn new stuff because of the neighborhood regeneration project.	
	INF3	I know the profile of other neighborhood regeneration projects nearby.	
Motivation	MOT1 (deleted)	My family anticipates my participation in the neighborhood regeneration project.	Zhang et al. (2015) and Zheng et al. (2018)
	MOT2	My neighbors anticipate my involvement in the neighborhood regeneration project.	
	MOT3	Nearly everyone in my vicinity has either taken part in or intends to participate in the neighborhood regeneration project.	
	MOT4	My neighbors have told me about the benefits of the neighborhood regeneration projects.	
Behavioral skills	BS1	I am competent enough to participate in the neighborhood regeneration project.	Tan et al. (2020), Tsamlag et al. (2020), and Wang et al. (2021)
	BS2	I have enough information to support my participation in the neighborhood regeneration project.	
	BS3	My contacts can help me solve the problems I encounter in the neighborhood regeneration project.	
Participation willingness	PW1	I am willing to learn about the regeneration process of our neighborhood.	Wang X. et al. (2018)
	PW2	I am willing to spend time being involved in the process of neighborhood regeneration.	
	PW3	I am willing to pay a reasonable fee for the neighborhood regeneration projects.	
Perceived local government support	PLGS1 (deleted)	I will seek help from the local government if I have some problems in the neighborhood regeneration project.	Tang (2020) and Wang (2022)
	PLGS2	The staff in the local government have a good attitude when solving problems.	
	PLGS3	The staff in the local government can provide services for me quickly.	
	PLGS4	I think the local government play an important role in neighborhood regeneration project.	
Participation behavior	PB1	I was involved in the discussion of the regeneration plan.	Du et al. (2020)
	PB2 (deleted)	I made compromises to get more residents involved in the neighborhood regeneration project.	
	PB3	I played an active role in convincing other residents to engage in the neighborhood regeneration project.	
	PB4	I have taken the initiative to learn about the relevant affairs of the neighborhood regeneration project.	

The survey took place from July 2022 and October 2022, targeting residents who currently reside in, are about to move to, or have previously experienced neighborhood regeneration projects. There were 565 participants in the survey, with 477 questionnaires deemed valid. Demographic data of the respondents is presented in Table 2. Of the total respondents, males accounted for 44.03% and females 55.97%. In terms of age, 47.80% of the respondents were over 50 years old, while only 9.85% were under 30 years old. Thus, most residents residing in dilapidated neighborhoods were middle-aged and elderly individuals. Additionally, 89.1% of the respondents had lived in their neighborhood for over 4 years, and 26.42% for more than 10 years.

**Table 2:** The demographic composition of the samples.

Variable	Categories	Frequency	Percent (%)
Gender	Male	210	44.03
	Female	267	55.97
Age	≤30	47	9.85
	31–50	202	42.35
	≥50	228	47.80
Living time (years)	≤3	52	10.90
	4–6	132	27.67
	7–9	167	35.01
	≥10	126	26.42

### 3.3. Structural equation modeling

Structural equation model (SEM) is a statistical method combining factor analysis and path analysis, which can deal with the relationship between the latent variable and their observable indicators (Byrne, 2010). SEM is especially advantageous for handling multi latent variables, which are difficult to be measured directly and accurately by traditional statistical methods (Kline, 2011). SEM is increasingly popular in neighborhood/urban regeneration research because it can be used to test correlations between variables and test management theoretical models (Wu et al., 2017; Wang and Xiang, 2019; Huang et al., 2022). In this study, SEM was used to explore the inherent mechanism of residents’ participation behavior based on the extended IMB model.

First, confirmatory factor analysis (CFA) was performed to test the fit and find an adequate measurement model. The item reliability, composite reliability, and convergence validity were examined (DeVellis and Thorpe, 2021). Second, the original IMB model was compared with the expanded IMB model, including the structural model. Both the measurement model and structural model were estimated via the maximum likelihood estimation. Model fit was assessed with the model chi-square ( $\chi^{2}$ ), the standardized root mean square residual (SRMR), the root mean square error of approximation (RMSEA), the Tucker and Lewis index (TLI), and the comparative fit index (CFI; Hair et al., 2010; Kline, 2011). Non-significant $\chi^{2}$ , values of 5 or smaller for $\chi^{2}/DF$ , 0.08 or below for the SRMR, equal to or less than 0.1 for the RMSEA, and above 0.09 for the CFI and TLI were generally considered a sign of good fit (Barrett, 2007; Byrne, 2010). The best-fitting model was reported for hypothesis testing (H1a~H1b, H2a~H2b, H3, H4a~H4b). The mediation effect of participation willingness was tested with the bootstrap approach (H5a~H5c).

## 4. Results and analyses

### 4.1. Validity and reliability of the measured constructs

As presented in the third column of Table 3, the measurement models exhibit a satisfactory fit outcome ( $\chi^{2}/DF=$ 3.365; SRMR = 0.050; RMSEA = 0.070; 90% CI [0.063–0.078]; TLI = 0.952; CFI = 0.962), as evidenced by the desirable ranges specified in column 2 (Chen and Knight, 2014; Wang Z. et al., 2017).

**Table 3:** The goodness of fit of models.

Parameters	Desirable range	CFA	SEM-IMB (BS)	SEM-IMB (PW)	SEM-IMB (BS + LRO)	SEM-IMB (PW + LRO)
			Model I	Model II	Model III	Model IV
$\chi^{2}$	/	403.751^***^	200.484^***^	192.994^***^	283.133^***^	280.570^***^
DF	/	120	48	48	80	80
$\chi^{2}/DF$	≤5.0	3.365	4.177	4.021	3.539	3.507
SRMR	≤0.08	0.050	0.050	0.053	0.050	0.053
RMSEA	≤0.10	0.070	0.082	0.080	0.073	0.072
RMSEA 90% CI	/	0.063–0.078	0.070–0.093	0.068–0.091	0.064–0.082	0.063–0.082
TLI	≥0.90	0.952	0.941	0.953	0.950	0.956
CFI	≥0.90	0.962	0.957	0.966	0.962	0.967

^***^p<0.001.

Due to the low loading estimates (i.e., FL *<* 0.50), two items, namely MOT1 and LRO1, were removed from this CFA to decrease measurement error (Hair et al., 2010; see Table 4). The factor loadings for all remaining factors are significant (*p* < 0.001), and they range from 0.698 to 0.969, exceeding a threshold value of 0.5. The results of the principal component analysis in Table 5 also showed good construct validity. In addition, the reliability of the IMB constructs ranged from good to acceptable, as indicated by their Cronbach’s alpha coefficients ( $\alpha_{I}=0.853,\alpha_{M}=0.759$ , $\alpha_{BS}=0.912$ , $\alpha_{EU}=0.960$ , $\alpha_{RGO}=0.951$ , $\alpha_{\mathrm{PB}}=0.856$ ). A coefficient between 0.50 to 0.70 denotes moderate reliability, while a coefficient exceeding 0.70 signifies excellent reliability (Hinton et al., 2014).

**Table 4:** Results of confirmatory factor analysis.

Constructs	Measures	Mean	FL
Information ( $\alpha_{{}_{I}}=$ 0.853)	INF1	4.681	0.924^***^
	INF2	4.474	0.846^***^
	INF3	4.472	0.709^***^
Motivation ( $\alpha_{{}_{M}}=$ 0.759)	MOT1 (deleted)	/	/
	MOT2	5.681	0.715^***^
	MOT3	5.591	0.744^***^
	MOT4	5.694	0.698^***^
Behavioral skills ( $\alpha_{{}_{BS}}=0.$ 912)	BS1	4.939	0.876^***^
	BS2	4.751	0.928^***^
	BS3	4.499	0.844^***^
Participation willingness ( $\alpha_{{}_{PW}}=0.$ 960)	PW1	5.551	0.969^***^
	PW2	5.465	0.934^***^
	PW3	5.597	0.928^***^
Perceived local government support ( $\alpha_{{}_{\mathrm{PlGS}}}=0.951$ )	PLGS1(deleted)	/	/
	PLGS2	4.730	0.946^***^
	PLGS3	4.589	0.970^***^
	PLGS4	4.369	0.888^***^
Participation behavior ( $\alpha_{{}_{\mathrm{PB}}}=0.856$ )	PB1	5.740	0.771^***^
	PB2	/	/
	PB3	4.839	0.856^***^
	PB4	5.136	0.827^***^

^***^p < 0.001. α means Cronbach’s alpha coefficient.

**Table 5:** Component analysis with varimax rotation.

	Component				
	1	2	3	4	5
INF1			0.897		
INF2			0.879		
INF3			0.714		
MOT2				0.741	
MOT3				0.841	
MOT4				0.727	
PLGS2		0.907			
PLGS3		0.924			
PLGS4		0.921			
PW1	0.855				
PW2	0.838				
PW3	0.827				
PB1					0.584
PB3					0.702
PB4					0.760

The table only contains loadings that were higher than 0.5; the rotation converged in 6 iterations.

Further, we also test the item reliability, composite reliability, convergence validity, and discriminate validity of the extended IMB model proposed in this paper. The results are shown in Table 6. There are 5 dimensions in total, and each dimension has 3 items. In our extended IMB model, the factor loading (FL) values for all the items are between 0.6 and 0.9, the values of construct reliability (CR) are all above 0.7, and average variance extracted (AVE) are all over 0.5. These values are within acceptable limits (Hair et al., 2010). In relation to discriminate validity, as suggested by Fornell and Larcker (1981), the diagonal of the matrix shows the square root of AVE, while the lower triangle represents the Pearson Correlation Coefficient between the dimensions. The comparison results show that the square root value is greater than the correlation of other related dimensions. Thus, our dimension has discriminant validity.

**Table 6:** Results of item reliability, construct reliability, convergence validity and discriminate validity.

Dimension	Item reliability	Construct reliability	Convergence validity	Discriminate validity				
	FL	CR	AVE	INF	MOT	PLGS	PW	PB
INF	0.704~0.930	0.869	0.690	0.831				
MOT	0.698~0.742	0.762	0.517	0.342	0.719			
PLGS	0.888~0.970	0.954	0.875	0.360	0.276	0.935		
PW	0.928~0.969	0.961	0.891	0.479	0.636	0.460	0.944	
PB	0.781~0.851	0.859	0.670	0.533	0.705	0.528	0.814	0.819

FL, factor loading; CR, construct reliability; AVE, average variance extracted.

### 4.2. Comparing the original and extended IMB model

**Figure 3:**
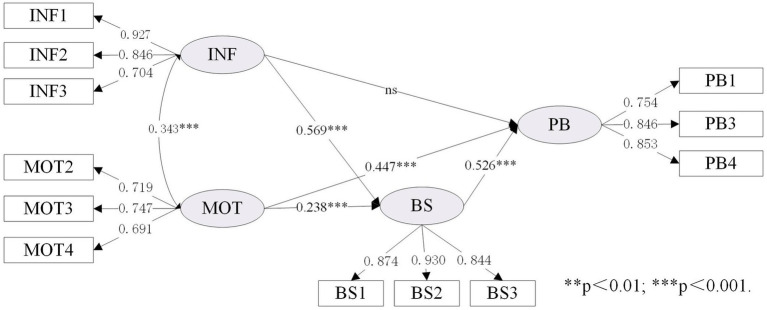
Standardized estimates of the IMB (BS) model.

**Figure 4:**
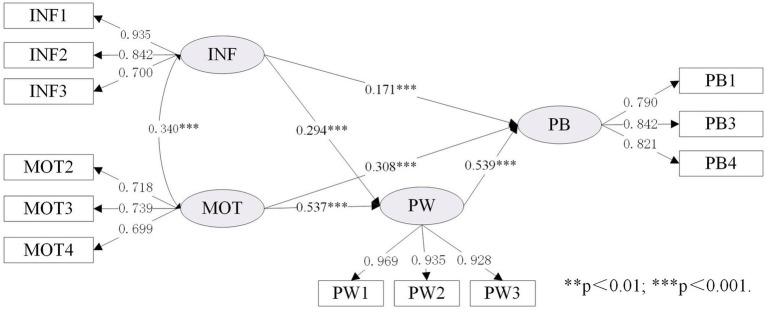
Standardized estimates of the IMB (PW) model.

**Figure 5:**
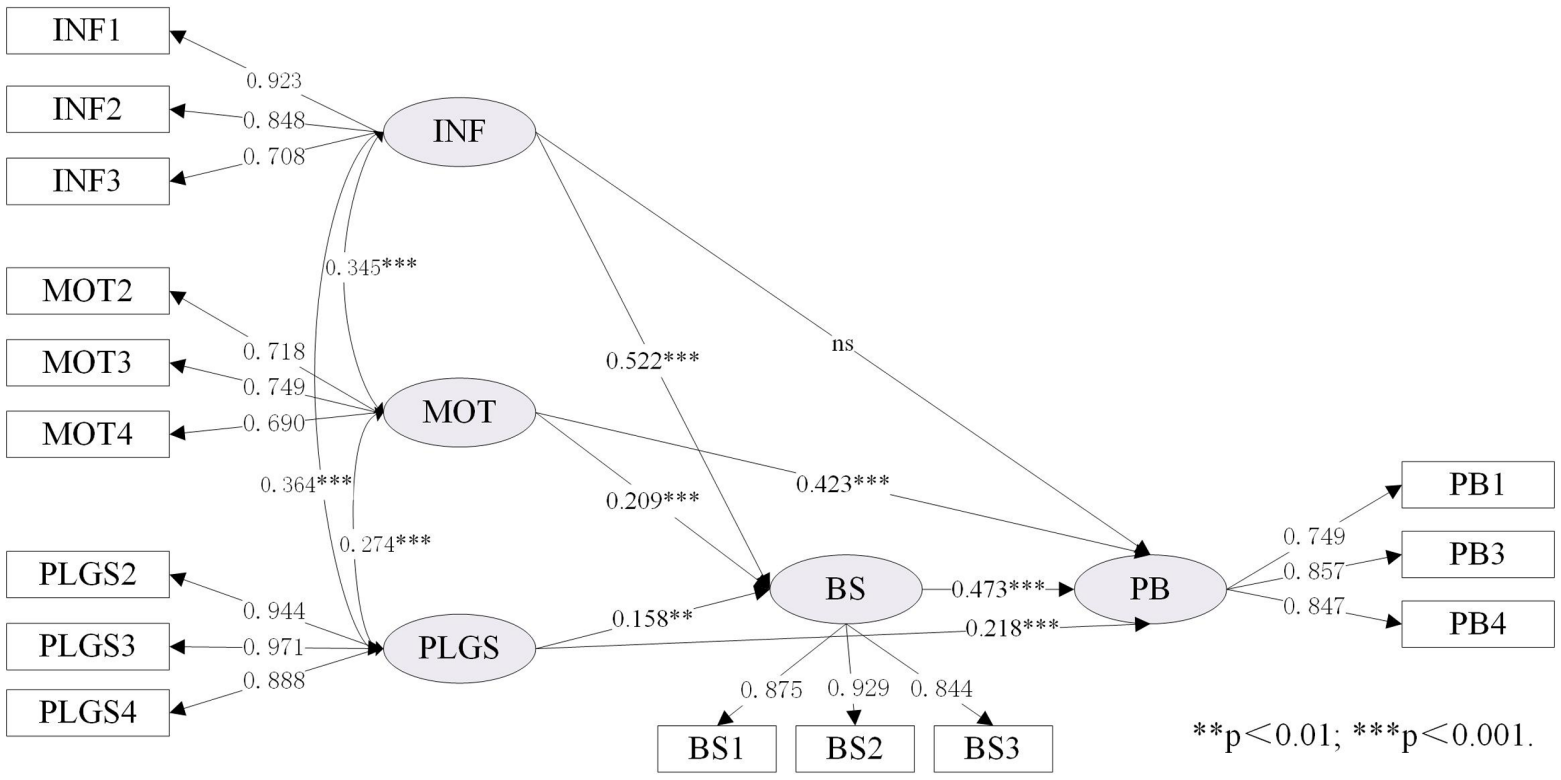
Standardized estimates of the IMB (BS + PLGS) model.

**Figure 6:**
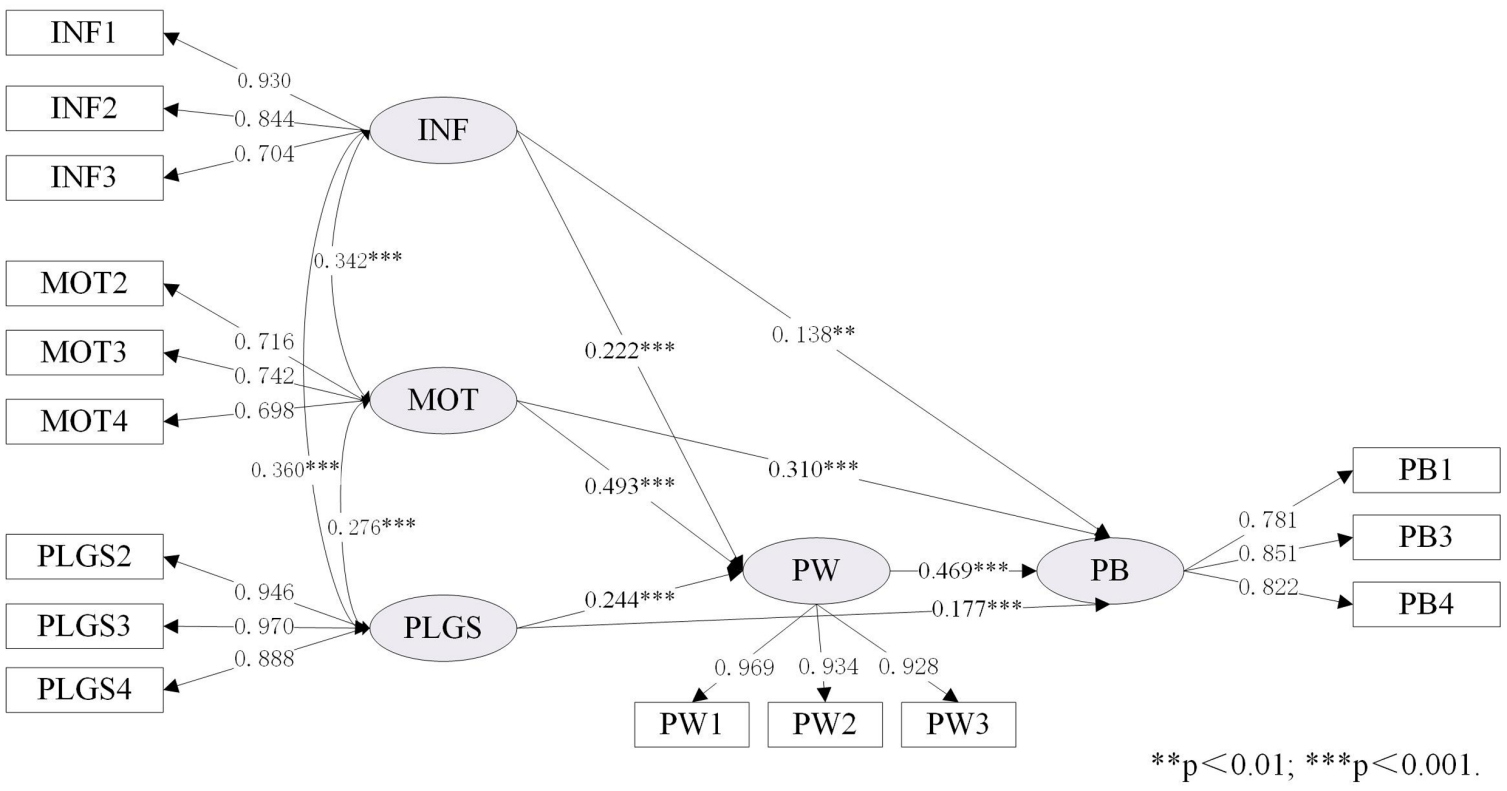
Standardized estimates of the IMB (PW + PLGS) model.

As shown in Table 7, the expanded IMB model (namely Model IV) was compared with the original IMB model (namely Model I). To better illustrate the differences before and after the IMB model expansion, we set up Model II and Model III to facilitate comparison. As shown in Table 3 (section 4.1), the data fitting results of the four models were all good, and the relevant fitting data of the expanded IMB model was performed as follows: $\chi^{2}/DF$ =3.507; SRMR = 0.053; RMSEA = 0.072, 90% CI [0.063–0.082]; TLI = 0.956; CFI = 0.967. Details of the standardized estimates for these four models are provided in Figures 3–6.

**Table 7:** Comparison of original and expanded IMB models.

Structural weight	IMB (BS)	IMB (PW)	IMB (BS + PLGS)	IMB (PW + PLGS)
	Model I	Model II	Model III	Model IV
BS ← INF	0.569^***^	/	0.522^***^	/
BS ← MOT	0.238^***^	/	0.209^***^	/
BS ← PLGS	/	/	0.158^**^	/
PW ← INF	/	0.294^***^	/	0.222^***^
PW ← MOT	/	0.537^***^	/	0.493^***^
PW ← PLGS	/	/	/	0.244^***^
PB ← INF	ns	0.171^***^	ns	0.138^**^
PB ← MOT	0.447^***^	0.308^***^	0.423^***^	0.310^***^
PB ← PLGS	/	/	0.218^***^	0.177^***^
PB ← BS	0.526^***^	/	0.473^***^	/
PB ← PW	/	0.539^***^	/	0.469^***^
BS (R2)	0.473	/	0.496	/
PW (R2)		0.482	/	0.532
PB (R2)	0.729	0.750	0.765	0.768

^**^p<0.01. ^***^p<0.001.

Table 7 showed the differences between the original IMB model and the extended IMB model, especially in terms of squared multiple correlation (R^2^). Comparing Model II with Model I, it was found that the value of R^2^ increased from 0.729 to 0.750, indicating that changing the BS construct to the PW construct helped to improve the overall explanatory power of the IMB model. Similarly, the comparison of Model III with Model I showed the need to add the LRO construct (the value of R^2^ increased from 0.729 to 0.765). It is therefore reasonable to speculate that the expanded IMB model has better explanatory power than the original IMB model. This is indeed the case, as shown in Table 7, where the R^2^ value for the expanded IMB model is 0.768, an increase of 0.039 compared to the R^2^ value for the original IMB model.

### 4.3. Hypothesis testing

#### 4.3.1. Direct effects

As shown in Figure 6, both INF (*β* = 0.138, *p* < 0.01), MOT (*β* = 0.310, *p* < 0.001) and PLGS (*β* = 0.177, *p* < 0.001) have a significant impact on PB, supporting H1a, H2a, and H4a. In addition, INF (*β* = 0.222, *p* < 0.001), MOT (*β* = 0.493, *p* < 0.001), and PLGS (*β* = 0.244, *p* < 0.001) also have a significant impact on PW, which can support H1b, H2b and H4b. Consistent with H3, PW (*β* = 0.469, *p* < 0.001) has a significant impact on PB.

#### 4.3.2. Mediation effects

The testing results of the mediation effect based on the bootstrapped confidence intervals were shown in Table 8. The mediation effects of INF (*β* = 104; SE = 0.029; 95% CI = [0.052, 0.162]), MOT (*β* = 0.231; SE = 0.034; 95% CI = [0.170, 0.306]), and PLGS (*β* = 0.115; SE = 0.026; 95% CI = [0.066, 0.172]) on PB via PW are both significant, supporting H5a, H5b, and H5c. Therefore, it can be concluded that residents’ participation willingness mediates the relationship between information, motivation, local government organizations on residents’ participation behavior.

**Table 8:** Testing of the mediation effects.

Path	Effect size	SE	BC bootstrap 95% CI	
			Lower	Upper
Path A: INF → PW → PB	0.104^***^	0.029	0.052	0.162
Path B: MOT → PW → PB	0.231^***^	0.034	0.170	0.306
Path C: PLGS → PW → PB	0.115^***^	0.026	0.066	0.172

^***^p < 0.001. SE, standard error.

## 5. Discussion

### 5.1. Contribution to the inherent mechanism of residents’ participation behavior

This study aims to explore the inherent relationship between individual, social and external factors and resident’s behavior based on the extended IMB model. The findings in this research suggest that replacing the “behavioral skills” construct with the “participation willingness” construct would improve the explanatory power of the IMB model in neighborhood regeneration and urban development research. A comparison of the original and extended IMB models in Table 7 (section 4.2) shows that the R^2^ value increased from 0.729 to 0.768, with a value of 0.021 for the change in R^2^ caused by the construct of “behavioral skills” to the construct of “participation willingness,” suggesting that the change in construct does facilitate an increase in explanatory power. In addition, a comparison of the models in Figures 5, 6 shows that the transformation of the “behavioral skills” construct to the “participation willingness” construct also changes the pathway of information influence on participation behavior from unsupportive to supportive. Indeed, the hypothesis that the influence pathway of “information-behavior” is not supported has emerged from previous research in many fields (Seacat and Northrup, 2010; Wang X. et al., 2018). This study found that changing the construct can make the direct path of information to the dependent variable significant, which may be an important theoretical contribution to improving the IMB model.

In addition, this study confirms that “participation willingness” plays an important mediating role between various factors and participation behavior. The importance of promoting participation willingness has been well documented in previous studies. Maitland (2006) pointed out how vital it is to grasp residents’ willingness and expectations to guarantee the effectiveness of their involvement. Some scholars noted that residents’ willingness has a strong influence on their participation and cooperation (Zhuang et al., 2017). However, most of these studies have used qualitative case studies, which make it difficult to clearly portray the inherent mechanism of residents’ participation behavior. This study provides a complementary exploration of the inherent mechanisms with the help of quantitative analysis methods (i.e., SEM). The research findings identify the mediating role of “participation willingness,” while elucidating the pathways in which various factors influence participation behavior, providing a theoretical contribution to gain insight of residents’ participation behavior in neighborhood regeneration projects.

Furthermore, this study also shows that the information available to individuals, the motivation influenced by neighbors, and the role of external government organization all have direct effects on residents’ participation willingness, with motivation having the greatest impact. Several scholars have discussed the influence of neighborhood effects on people’s behavior. Neighborhoods are often viewed as groups of residents who share similar normative values and practices, making them appear homogenous (Dalton, 2008; Huang and Pai, 2015). If there is a participation style that is formed and preferred by a group of residents, this may increase the willingness of other residents to participate in regeneration using the same style (Dodoiu, 2015; Xu et al., 2017). This means that residents’ participation willingness and the implementation of their behavior will be heavily influenced by their families, neighbors and surroundings (Zhang et al., 2015). Particularly in older neighborhoods, where residents have long-established social relationships, these social pressures may drive changes in residents’ willingness and behavior (Wang C. et al., 2018).

### 5.2. The important role of the local government organization

A consensus has been gradually formed in which “public participation became a subject of public policy” and in which key stakeholders of civil society were engaged in the neighborhood and urban regeneration (Degen and García, 2012). However, when it comes to making decisions on complex public issues, it is still questionable whether residents are fully rational actors (Fung, 2006). Due to the relatively low level of knowledge regarding urban development among residents, it is sometimes difficult for most of them to make sound decisions on complex professional issues on their own (Liu et al., 2021). Hence, there is a suggestion that government organizations should play a guiding and assisting role in resident participation (Baek and Kwon, 2020). In China in particular, the current state of civil society is still in its infancy (Verdini, 2015). Residents have become accustomed to relying on the government for a long time and even believe that it is the governments’ obligation to improve their living environment. Hence, to promote the function of civil society for effective residents’ participation, the local government organizations need to play a role in supporting resident interests and public needs when participating in the neighborhood regeneration process (Chen and Qu, 2020).

Some scholars have examined the interaction between government organizations and residents’ behaviors. For example, Li and Zhu (2014) developed a conceptual framework for decision-making to foster innovative public participation mechanisms. Similarly, Li X. et al. (2020) investigated residents’ involvement in urban heritage management in Lijiang, China, and highlighted the crucial role of local governmental organizations in guiding residents’ engagement in public affairs. Our study examines the role played by local government organizations in the inherent mechanism of residents’ participation behavior in neighborhood regeneration projects, providing a quantitative study as a supplement. The findings show that it has a significant direct effect on both residents’ participation willingness and their participation behavior, which can provide insights for future policy development. On the one hand, the government organizations need to use a variety of innovative tools to promote residents’ willingness to participate, and on the other hand, they need to provide channels for residents’ participation behavior.

### 5.3. Limitations and future research

The present study has some limitations. First, similar to sociological surveys of civic participation in general, it was inevitable that our sample consisted of self-selected residents. Participation in the questionnaire study can also be considered, to some extent, as an act of participation behavior. It is reasonable to assume that those who are willing to participate in our study are more likely to be involved in the neighborhood regeneration projects than those who refuse to participate. In-depth interviews and questionnaires were also deliberately conducted with residents of the lower floors of the staircase extension (the group of residents usually considered to be the least agreeable to the regeneration), so our research sample covered a wide range of people and was able to provide rich support for our findings and conclusions. Second, the empirical research in this paper confirms that local government organizations do have an impact on residents’ participation willingness and behavior in neighborhood regeneration projects in the Chinese context, but further research is needed on what specific measures government organizations should take in practice.

## 6. Conclusion

The aim of this study is to explore the inherent mechanisms of residents’ participation behavior in neighborhood regeneration projects. Three aspects of influencing factors (individual, social and external macro-environment) were identified, and the interaction of these influencing factors were clarified. In this study, SEM was used to test the hypotheses of the extended IMB model. The results show that all the research hypotheses are valid and that the expanded IMB model has stronger explanatory power than the initial model. This study also shows that the information available to individuals, the motivation influenced by neighbors, and the role of external government organization all have an impact on residents’ participation willingness, with motivation having the greatest one. Secondly, the study found that local government organizations have a direct impact on both residents’ willingness and their behavior, implying that government organizations can take innovative initiatives to promote residents’ willingness to participate (e.g., by using the motivational effect of resident interaction), while providing channels of participation to facilitate the shift from willingness to behavior. The findings and conclusions of this study are conducive to enriching the theoretical system of residents’ participation in neighborhood and urban regeneration, and providing scientific references for the urban development authorities to formulate policies.

## Data availability statement

The raw data supporting the conclusions of this article will be made available by the authors, without undue reservation.

## Author contributions

XF: Conceptualization, Data curation, Methodology, Writing – original draft. TZ: Data curation, Resources, Writing – review & editing. RH: Conceptualization, Writing – review & editing. YD: Resources, Supervision, Writing – review & editing.
